# Ergot in the Treatment of Glycosuria

**Published:** 1882-10

**Authors:** 


### ORIGINAL TRANSLATIONS AND ABSTRACTS.

### ERGOT IN THE TREATMENT OF GLYCOSURIA.

Dr. E. F. Tiedemann (*Medical News*, July 22, '82) has reported a case of diabetes mellitus, in which the most surprising effects followed the administration of half-dram doses of ergot combined with fifteen grains salicylic acid, twenty grains sodium bicarbonate, and ten minims of tincture of opium every four hours. The usual strict diet of proteids and fats was also ordered.

After four doses of the medicine had been taken, the quantity of urine voided in the twenty-four hours fell from twenty pints to eight pints. The specific gravity had only increased from 10.34 to 10.36 in the same time. In two months the average daily discharge of urine was about five pints, with specific gravity varying from 10.27 to 10.30. The patient had regained his former strength and could work as hard as ever. The ergot was continued twice a day. At one time, two weeks after beginning treatment, the daily discharge of urine fell to two pints.

This treatment of a hitherto very intractable disease certainly merits trial.

